# Correction: Incidence, clinical course and risk factor for recurrent PCR positivity in discharged COVID-19 patients in Guangzhou, China: A prospective cohort study

**DOI:** 10.1371/journal.pntd.0010032

**Published:** 2021-12-10

**Authors:** Jiazhen Zheng, Rui Zhou, Fengjuan Chen, Guofang Tang, Keyi Wu, Furong Li, Huamin Liu, Jianyun Lu, Jiyuan Zhou, Ziying Yang, Yuxin Yuan, Chunliang Lei, Xianbo Wu

There are errors in the Funding statement. The correct Funding statement is as follows: This study was supported by the Open Project of Guangdong Provincial Key Laboratory of Tropical Disease Research (G818330002). The funder had no role in study design, data collection and analysis, decision to publish, or preparation of the manuscript.

## References

[1] Zheng J, Zhou R, Chen F, Tang G, Wu K, Li F, et al. (2020) Incidence, clinical course and risk factor for recurrent PCR positivity in discharged COVID-19 patients in Guangzhou, China: A prospective cohort study. PLoS Negl Trop Dis 14(8): e0008648. 10.1371/journal.pntd.0008648 32866168 PMC7505432

