# Supplementary material for: Incidence, clinical course and risk factor for recurrent PCR positivity in discharged COVID-19 patients in Guangzhou, China: A prospective cohort study
Source: PLoS Negl Trop Dis. 2020 Aug 31;14(8):e0008648. doi: 10.1371/journal.pntd.0008648 (PMC7505432; doi:10.1371/journal.pntd.0008648)
Supplement: S1 Table — Data of the indicators that have been measured many times are median (IQR) value of first three weeks after admission. Others are data at admission. In the first, second and third weeks, the number of available test results of RP patients were 27, 27, 19, in contrast, 258, 249, 184 in NRP patients. P values comparing RP and NRP patients are from Mann-Whitney U test. GFR = glomerular filtration rate. ALT = Alanine aminotransferase. AST = Aspartate aminotransferase. RP = redetectable as positive. NRP = non-redetectable as positive. (DOCX) [file pntd.0008648.s001.docx]

**S1 Table. Laboratory findings for RP and non-RP patients.**

| **Parameter** | **Normal Range** | **All patients** | **RP patients** | **NRP patients** | **p value** |
| --- | --- | --- | --- | --- | --- |
| CD45+, μg/L | 1488-4483 | 1446.0 (1126.0-1789.0) | 1440.0 (1195.0-1631.0) | 1451.0 (1103.0-1815.0) | 0.708 |
| First week |  | 1262.0 (1144.0-1789.0) | 1195.0 (1144.0-1815.0) | 1321.0 (1017.0-1789.0) | 1.000 |
| Second week |  | 1343.0 (805.0-1770.0) | 1292.0 (1250.0-1334.0) | 1414.0 (790.0-2090.0) | 0.655 |
| Third week |  | 1439.0 (1103.0-1631.0) | 1523.0 (1087.0-1675.0) | 1424.0 (1103.0-1629.0) | 0.836 |
| CD3+CD45+, μg/L | 955-2860 | 1008.5 (757.5-1233.5) | 1064.0 (754.0-1272.0) | 1002.0 (761.0-1221.0) | 0.615 |
| First week |  | 996.0 (754.0-1339.0) | 999.0 (754.0-1289.0) | 987.0 (677.0-1368.5) | 1.000 |
| Second week |  | 1000.0 (616.8-1274.5) | 1054.5 (980.0-1129.0) | 1000.0 (601.5-1351.0) | 0.858 |
| Third week |  | 1002.5 (774.0-1188.5) | 1119.0 (810.5-1315.5) | 994.0 (774.0-1149.0) | 0.340 |
| CD3+CD4+, μg/L | 550-1440 | 583.5 (418.0-719.0) | 564.0 (397.0-692.0) | 592.0 (425.0-722.0) | 0.526 |
| First week |  | 500.0 (385.0-647.0) | 396.0 (385.0-648.0) | 502.0 (387.8-632.0) | 0.773 |
| Second week |  | 542.8 (339.5-788.5) | 462.5 (424.0-501.0) | 585.3 (334.0-800.0) | 0.593 |
| Third week |  | 612.8 (426.0-711.0) | 593.0 (471.0-708.5) | 612.8 (426.0-711.0) | 0.968 |
| Monocyte, ×10^9^ /L | 0.1-0.6 | 0.4 (0.3-0.5) | 0.4 (0.3-0.4) | 0.4 (0.3-0.5) | 0.923 |
| First week |  | 0.4 (0.3-0.5) | 0.4 (0.3-0.5) | 0.4 (0.3-0.5) | 0.502 |
| Second week |  | 0.4 (0.3-0.5) | 0.4 (0.3-0.4) | 0.4 (0.3-0.5) | 0.334 |
| Third week |  | 0.4 (0.3-0.5) | 0.4 (0.3-0.5) | 0.4 (0.3-0.5) | 0.767 |
| Alanine aminotransferase, U/L | 9-50 | 21.9 (15.9-32.0) | 19.7 (14.5-30.8) | 22.0 (16.0-32.0) | 0.521 |
| First week |  | 20.3 (14.0-30.9) | 19.7 (13.9-33.7) | 20.4 (14.1-30.0) | 0.950 |
| Second week |  | 21.4 (15.0-38.2) | 17.1 (14.8-33.7) | 22.2 (15.3-39.7) | 0.315 |
| Third week |  | 25.4 (16.6-36.5) | 21.9 (13.7-36.1) | 26.0 (17.4-36.7) | 0.333 |
| Aspartate aminotransferase, U/L | 15-40 | 18.7 (15.4-23.7) | 17.4 (15.2-20.6) | 18.7 (15.4-24.1) | 0.317 |
| First week |  | 19.8 (16.2-27.5) | 19.0 (15.8-24.0) | 19.8 (16.4-27.8) | 0.395 |
| Second week |  | 17.5 (14.2-22.3) | 15.2 (14.4-19.6) | 17.5 (14.0-23.2) | 0.258 |
| Third week |  | 17.9 (14.3-23.1) | 15.8 (14.6-19.7) | 18.1 (14.3-23.3) | 0.207 |
| GFR, mL/min/1.73 m^2^ | 90-120 | 132.2 (110.6-154.8) | 138.7 (116.9-161.1) | 131.9 (109.2-151.9) | 0.496 |
| First week |  | 137.6 (113.2-162.0) | 138.7 (121.1-164.2) | 137.6 (111.7-161.0) | 0.614 |
| Second week |  | 126.6 (108.1-147.5) | 121.4 (105.9-140.8) | 126.9 (108.6-148.6) | 0.698 |
| Third week |  | 124.4 (104.4-143.6) | 123.9 (104.8-147.6) | 125.4 (104.4-143.3) | 0.900 |
| PaO_2_, mmHg | 83-108 | 96.1 (83.0-113.0) | 92.3 (83.2-107.0) | 96.1 (83.0-115.0) | 0.508 |
| First week |  | 93.0 (79.6-107.1) | 92.4 (83.2-104.0) | 93.1 (79.3-108.0) | 0.973 |
| Second week |  | 104.0 (85.5-133.4) | 107.0 (86.0-111.0) | 103.0 (84.4-137.0) | 0.921 |
| Third week |  | 101.0 (86.8-123.8) | 100.0 (87.5-110.7) | 101.0 (86.8-126.0) | 0.635 |
| Hemoglobin, g/L | 115-150 | 136.0 (123.5-146.0) | 137.0 (125.0-143.0) | 136.0 (123.0-146.0) | 0.937 |
| Red blood cell, × 10^9^ per L | 3.8-5.1 | 4.4 (4.1-4.8) | 4.5 (4.3-4.8) | 4.4 (4.1-4.8) | 0.230 |
| Hematocrit, % | 35-45 | 40.1 (36.5-42.6) | 40.0 (38.5-41.6) | 40.1 (36.4-42.6) | 0.947 |
| Platelet count, × 10^9^ per L | 125-350 | 186.5 (150.5-231.5) | 198.0 (163.0-231.0) | 186.0 (148.0-232.0) | 0.528 |
| Albumin, g/L | 40-55 | 40.1 (36.7-42.5) | 41.8 (38.9-44.7) | 39.9 (36.2-42.3) | 0.005 |
| Total bilirubin, mmol/L | 0-21 | 9.6 (6.7-13.8) | 10.9 (8.8-20.0) | 9.5 (6.7-13.6) | 0.118 |
| Potassium, mmol/L | 3.4-4.5 | 0.09 (0.04-30.0) | 3.6 (3.3-3.8) | 3.6 (3.3-3.9) | 0.766 |
| Sodium, mmol/L | 136-146 | 140.0 (138.0-142.0) | 140.6 (140.0-143.0) | 140.0 (138.0-142.0) | 0.100 |
| Chlorine, mmol/L | 98-106 | 105.0 (103.0-107.0) | 105.0 (103.0-108.0) | 105.0 (103.0-106.0) | 0.422 |
| Anion gap, mmol/L | 10-14 | 10.2 (8.9-11.2) | 10.6 (9.4-12.0) | 10.2 (8.9-11.2) | 0.193 |
| Blood PH | 7.35-7.45 | 7.39 (7.37-7.42) | 7.39 (7.37-7.41) | 7.39 (7.37-7.42) | 0.848 |
| PaCO_2_, mmHg | 35-45 | 41.0 (38.1-44.0) | 41.6 (39.5-43.3) | 40.8 (38.0-44.5) | 0.679 |
| Blood oxygen saturation, % | 92-98 | 98.0 (96.9-98.8) | 98.0 (97.0-99.0) | 98.0 (96.9-98.7) | 0.457 |
| Respiratory index, % | 0-10 | 21.0 (5.0-51.0) | 14.5 (4.5-24.5) | 22.0 (5.0-51.0) | 0.235 |
| B-type Natriuretic Peptide, pg/ml | 0-400 | 34.0 (11.0-61.0) | 17.0 (12.0-27.0) | 35.5 (10.5-62.5) | 0.581 |
| Myoglobin, μg/L | 14.3-65.8 | 14.9 (8.9-22.1) | 15.4 (11.8-18.5) | 14.8 (8.6-22.2) | 0.665 |
| Troponin I, μg/L | 0-0.03 | 0.004 (0.001-0.009) | 0.005 (0.001-0.038) | 0.004 (0.001-0.009) | 0.838 |
| Urine specific gravity | 1.003-1.030 | 1.018 (1.013-1.022) | 1.018 (1.013-1.021) | 1.018 (1.013-1.023) | 0.666 |
| Urine leukocyte, P/μl | 0-12 | 2.9 (0.9-8.5) | 1.5 (0.0-5.9) | 2.9 (0.9-8.9) | 0.037 |
| Urinary Erythrocytes, P/μl | 0-10 | 1.3 (0.0-3.9) | 1.3 (0.0-2.9) | 1.3 (0.0-5.3) | 0.370 |
| Urea, mmol/L | 2.6-8.8 | 3.8 (3.2-4.5) | 3.7 (3.4-4.5) | 3.8 (3.1-4.6) | 0.563 |
| Uric acid, μmol/L | 155-357 | 284.2 (228.8-365.0) | 309.0 (248.9-413.0) | 282.8 (225.3-362.9) | 0.126 |
| Total bilirubin, μmol/L | 0-21 | 9.6 (6.7-13.8) | 10.9 (8.8-20.0) | 9.5 (6.7-13.6) | 0.118 |
| AST/ALT | 0.8-1.5 | 1.0 (0.8-1.3) | 0.9 (0.7-1.3) | 1.0 (0.8-1.3) | 0.264 |
| Total protein, g/L | 65-85 | 68.4 (65.0-71.3) | 70.6 (66.9-72.1) | 68.0 (64.8-71.1) | 0.037 |
| Globulin, g/L | 20-40 | 28.6 (26.1-30.8) | 29.0 (25.9-30.1) | 28.5 (26.2-30.9) | 0.432 |
| Plasma prothrombin time, s | 11-15 | 13.5 (13.1-14.0) | 13.4 (13.0-13.6) | 13.5 (13.1-14.0) | 0.138 |
| Thrombin time, s | 13-21 | 16.1 (15.5-16.9) | 16.3 (15.6-17.0) | 16.1 (15.5-16.9) | 0.355 |
| Plasma fibrinogen, g/L | 2.0-4.0 | 3.7 (2.9-4.5) | 3.1 (2.7-4.0) | 3.8 (3.0-4.7) | 0.082 |
| Creatine kinase isoenzyme, U/L | 0-24 | 10.9 (8.9-14.6) | 10.7 (8.5-13.6) | 11.0 (9.1-14.8) | 0.437 |

Data of the indicators that have been measured many times are median (IQR) value of first three weeks after admission. Others are data at admission. In the first, second and third weeks, the number of available test results of RP patients were 27, 27, 19, in contrast, 258, 249, 184 in NRP patients. P values comparing RP and NRP patients are from Mann-Whitney U test. GFR=glomerular filtration rate. ALT=Alanine aminotransferase. AST=Aspartate aminotransferase. RP=redetectable as positive. NRP=non-redetectable as positive
