# Supplementary material for: Incidence, clinical course and risk factor for recurrent PCR positivity in discharged COVID-19 patients in Guangzhou, China: A prospective cohort study
Source: PLoS Negl Trop Dis. 2020 Aug 31;14(8):e0008648. doi: 10.1371/journal.pntd.0008648 (PMC7505432; doi:10.1371/journal.pntd.0008648)
Supplement: S2 Table — Data are median (IQR) or n (%). *Results of CT scan for first admission were shown in Table 1. **Only 20 RP patients have tested for Antibody. Results of Ct values and CD cell were median value during hospitalization. RP = redetectable as positive. NRP = non-redetectable as positive. (DOCX) [file pntd.0008648.s002.docx]

**S2 Table. Clinical characteristics, treatment and laboratory findings of 27 RP patients at first admission and readmission**

| **Characteristics** | **First-admission** | **Readmission** | **p value** |
| --- | --- | --- | --- |
| Hospitalization, days | 18.0 (13.0-24.0) | 7.0 (5.0-11.0) | <0.001 |
| **Severity** |  |  |  |
| Mild | 3 (11.1) | 20 (74.1) | <0.001 |
| Moderate | 24 (88.9) | 7 (25.9) | .. |
| **Symptoms** |  |  |  |
| Asymptomatic | 5 (18.5) | 17 (62.9) | 0.013 |
| Fever | 18 (66.7) | 1 (3.7) | .. |
| Dry cough | 14 (51.6) | 6 (22.2) | .. |
| Myalgia | 1 (3.7) | 2 (7.4) | .. |
| Expectoration | 6 (22.2) | 2 (7.4) | .. |
| Pharyngalgia | 0 | 1 (3.7) | .. |
| Dizziness | 2 (7.4) | 1 (3.7) | .. |
| Headache | 1 (3.7) | 1 (3.7) | .. |
| **Treatment** |  |  |  |
| Oxygen inhalation | 17 (62.9) | 8 (29.6) | .. |
| Antiviral therapy | 23 (85.2) | 1 (3.7) | .. |
| Antibiotic therapy | 20 (74.1) | 3 (11.1) | .. |
| Hydrogen and oxygen atomizer | 0 (0) | 1 (3.7) | .. |
| Traditional Chinese medicine | 17 (62.9) | 5 (18.5) | .. |
| Hormone | 4 (14.8) | 0 | .. |
| **Laboratory findings** |  |  |  |
| Ct values (N gene) | 35.0 (33.0-37.0) | 37.5 (36.0-38.5) | 0.042 |
| Ct values (ORF gene) | 35.0 (32.0-38.0) | 40.0 (37.0-40.0) | 0.065 |
| Antibody for SARS-CoV-2^**^ |  |  |  |
| IgG | 20 (100.0) |  |  |
| IgM | 16 (80.0) |  |  |
| CD45+ | 1440.0 (1144.0-1719.0) | 1555.0 (1195.0-1696.0) | 0.273 |
| CD3+ CD45+ | 1047.0 (754.0-1289.0) | 942.0 (842.0-1076.0) | 0.237 |
| CD3+ CD4+ | 564.0 (396.0-692.0) | 553.0 (460.0-692.0) | 0.484 |
| CD3+ CD8+ | 433.0 (324.0-592.0) | 346.0 (311.0-505.0) | 0.499 |

Data are median (IQR) or n (%). ^*^Results of CT scan for first admission were shown in Table 1. **^**^**Only 20 RP patients have tested for Antibody. Results of Ct values and CD cell were median value during hospitalization. RP=redetectable as positive. NRP=non-redetectable as positive.
