# Supplementary figures and images for: Incidence, clinical course and risk factor for recurrent PCR positivity in discharged COVID-19 patients in Guangzhou, China: A prospective cohort study
Source: PLoS Negl Trop Dis. 2020 Aug 31;14(8):e0008648. doi: 10.1371/journal.pntd.0008648 (PMC7505432; doi:10.1371/journal.pntd.0008648)

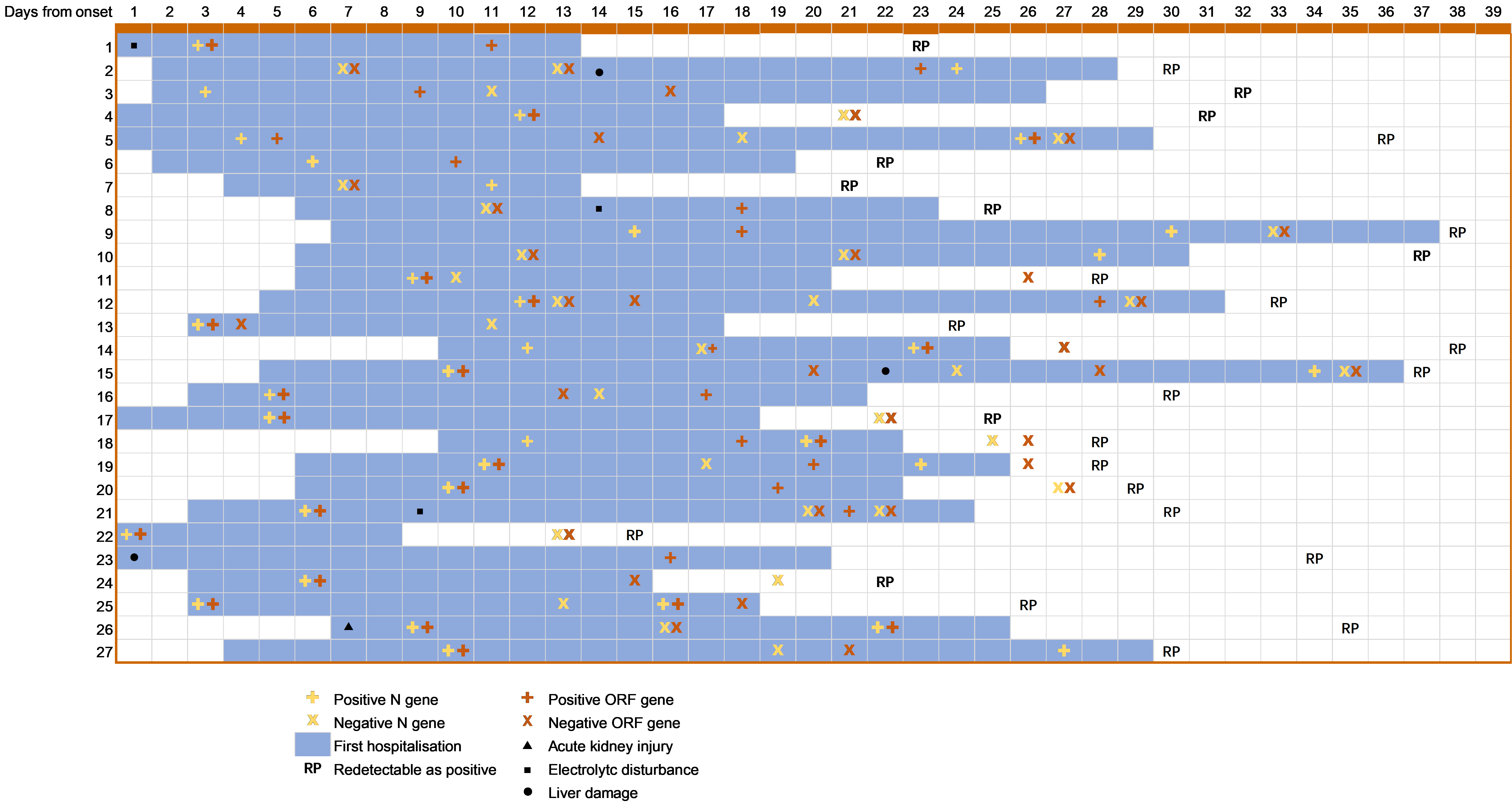

Supplement: S1 Fig — (TIF) [file pntd.0008648.s003.tif]
