# Supplementary material for: Incidence, clinical course and risk factor for recurrent PCR positivity in discharged COVID-19 patients in Guangzhou, China: A prospective cohort study
Source: PLoS Negl Trop Dis. 2020 Aug 31;14(8):e0008648. doi: 10.1371/journal.pntd.0008648 (PMC7505432; doi:10.1371/journal.pntd.0008648)
Supplement: Attachment — Submitted filename: detailed list of responses.docx [file pntd.0008648.s004.docx]

**List of Responses**

Dear Editors and Reviewers:
Thank you for your letter and for the reviewers’ comments concerning our manuscript entitled “Incidence, clinical course and risk factor for recurrent PCR positivity in discharged COVID-19 patients in Guangzhou, China: a prospective cohort study” (ID: ID: PNTD-D-20-00893). Those comments are all valuable and very helpful for revising and improving our paper, as well as the important guiding significance to our researches. We have studied comments carefully and have made correction which we hope meet with approval. Revised portion are marked in red in the paper. The main corrections in the paper and the responds to the reviewer’s comments are as following:

**Response to Reviewer 1:**

1.[ Major concern is this being a prospective study, informed consent is required as patient population was tested post discharge and the main criteria for inclusion in the study was RP positive. Manuscript mentions the requirement was waived by ethics board. PLEASE EXPLAIN WHY]

【Response】:We are very sorry for we haven’t clarified that it’s a routine for Guangzhou Eighth People's Hospital to perform viral nucleic acid detection on COVID-19 patients every two or three days during their post-discharge surveillance (within 15 days after discharge). Patients with positive nucleic acid tests were diagnosed as being RP and should be readmitted and receive further medical observation. Thus, the institutional ethics board granted a waiver for informed consent from the study patients.

【Change】

Line 89, the statement of “During post-discharge surveillance (within 15 days after discharge), nasopharyngeal swab samples of patients were collected by staff of Guangzhou Center for Disease Control and Prevention (Guangzhou CDC) and submitted to Guangzhou Eighth People's Hospital for Reverse transcription polymerase chain reaction (RT-PCR) test.” was corrected as “According to the COVID-19 management routine of Guangzhou Eighth People's Hospital, after initial discharging, COVID-19 patients have to undergo a period of isolation (within 15 days after discharge, it is unlikely to have a RP after this time) in the hospital or at home. During the surveillance, nasopharyngeal swab samples of patients were collected by staff of Guangzhou Center for Disease Control and Prevention (Guangzhou CDC) and submitted to Guangzhou Eighth People's Hospital for Reverse transcription polymerase chain reaction (RT-PCR) test.”

2.[ I can understand the CT images included for three RP patients and one NRP patient. But the relevance and clinical significance need to be explained. Review of literature shows very little correlation between imaging and patient clinical improvement. Even though the findings of eosinophil count LDH appear to have more clinical relevance in follow up too.]

【Response】: In a previous study, SARS-CoV-2 was found in lung cells in a COVID-19 patient with three consecutive negative PCR tests of nasopharynx swab samples which may be a reason for the RP [1]. Whether RP patients will recur pulmonary inflammation attract great concern. By displaying CT findings, we aimed to give a detailed description on the lung condition of RP patients and further confirm their recovery of pneumonia. And we found favorable inflammatory absorption in RP patients’ CT image. According to reviewer’s comment, we have supplemented relevance and clinical significance of the CT findings in the discussion. It’s a limitation that we did not further explore the impact of eosinophil count LDH because the laboratory finding of readmission was less comprehensive than the first time.

[1] Yao X-H, He Z-C, Li T-Y, Zhang H-R, Wang Y, Mou H, et al. Pathological evidence for residual SARS-CoV-2 in pulmonary tissues of a ready-for-discharge patient. Cell Res. 2020. doi:10.1038/s41422-020-0318-5

【Change】

Line 215, “When we focus on chest CT rather than viral load, most of the RP patients seem to be normal convalescent patients with favorable inflammatory absorption. For these patients, unless there is a clinical symptom worsening, excess clinical intervention may not be necessary.” was added.

3. [My concern is with the conclusion that these RP patients unlikely are infectious. Even though a considerable conclusion can be made that they are unlikely reinfected based on provided biochemical data and symptoms, we have no evidence that they are not infectious to other population or health care personnel. Please explain]

【Response】: The reviewer’s mention is reasonable and we apologize for our negligence. Despite no individual was infected by RP patients in this study, as post-discharge patients were under isolation, we were unable to effectively assess the infectiousness of RP patients. According to the Reviewer’s comment, the following corrections was made.

【Change】

1.Line 38 “may not represent infectious virus” was deleted.

2.Line 262-264, the statement of “It was observed these patients had returned to true negative soon, and none of them remained infectious; still, prolonged hospitalization for patients with lower efficiency in virus clearance might be a safer measure.” was corrected as “It was observed these patients had returned to true negative soon but their potential infectiousness remained unknown; thus, prolonged hospitalization for patients with lower efficiency in virus clearance might be a safer measure.”

3. Line 246, “Fourth, despite no individual was infected by RP patients in this study, as post-discharge patients were under isolation, we were unable to effectively assess the infectiousness of RP patients.” was added.

4.[Please explain the diverse characteristics of the adult population, which was described in the manuscript that makes the study representative.]

【Response】: According to the Reviewer’s comment, we found that the statement of “diverse characteristics” was confusing. We tried to express that the population was representative since we have included the majority of COVID-19 patients in Guangzhou, an international city, and there were no restrictions other than age.

【Change】

Line 206, the statements of “The heterogeneity of these results is probably due to the disparities in the sample size and population characteristics. The incidence rate of our study is representative due to the diverse characteristics of the study population.” were corrected as “The study by Zhongnan Hospital merely included medical staff and the Third People’s Hospital of Shenzhen had only enrolled 49.4% (209/423) COVID-19 patients diagnosed in Shenzhen. The incidence rate of our study was more representative since we have included the majority (75.6%, 285/377) of COVID-19 patients in the international city: Guangzhou, with no restrictions other than age.”

**Response to Reviewer 2:**

1.[Table 2 Normal range for CT value ≥40 needs to be defined, as per my understanding it should be ≤40, if the authors mean something else they should explain it in the footnotes.]

【Response】: Thank you for your comment. The diagnostic criteria in this study were based on the recommendation by the National Institute for Viral Disease Control and Prevention (China). Threshold refers to the critical value of fluorescence signal in exponential growth period. Cycle threshold value (Ct value) refers to the number of cycles when the fluorescence signal reaches the threshold. A Ct-value less than 37 was defined as positive, a Ct-value ≥40 was defined as negative. A medium load (Ct-value 37-40) was an indication for retesting. Lower Ct value refers to higher viral load.

2.[There is a difference in the viral shedding days between the N gene and ORF gene, can the authors postulate a probable cause for this phenomenon.]

【Response】: The difference in the viral shedding days between the N gene and ORF gene could possibly be explained by the difference in their sensitivity to nucleic acid detection. We are very sorry that we were unable to postulate a more definite cause for this phenomenon which needs further virological research.

3.[Line 168 results section mentions about the immunological assay (IgG and IgM) theses need to be defined in the methodology (test kit used, type of assay I,e, ELISA based or ICT).]

【Response】: Thank you for this kind guidance. We have added this point in line 107.

【Change】

Line 105, “COVID-19 Human IgM IgG Assay Kit (ELISA based, produced by Abnova) was used to test the IgG and IgM level in COVID-19 patients.” was added.

4.[Table 2 shows a p value of 0.045 for difference in Ct value at 3rd week however the results in table 4 are completely different. If both are comparison between RP and NRP then why this discrepancy. If these are two different analysis the title of the table should define it accordingly.]

【Response】: Thank you for this kind remind. We have checked the results and found no mistakes in these two results. The discrepancy mainly results from the two different analysis. Table 2 used Mann-Whitney test and Table 4 used single factor Logistic regression. We consider the difference between these two p value may still be within the acceptable range. According to the reviewer’s comment, we have supplemented the headings of Table 4.

【Change】

Line 187, “Risk factors for RP events” was corrected as “Univariable and multivariable analysis of risk factors associated with RP events”.

5.[Line 198-202 the language needs to be modified it sounds a bit confusing]

【Response】: Due to other modifications, the line number was changed to 203-206

【Change】

Line 202, the statement “27 of 285 (9.5%) individuals had tested positive for SARS-CoV-2 in nasopharyngeal swab after discharged which was higher than the rate reported by the study of Zhongnan Hospital, in which has detected two (3·23%) positive cases in recovered medical staff. Tang et al reported among 209 discharged patients, 9 (4.3%) re-tested positive in throat swabs only, 13 patients (6.2%) re-tested positive in anal swabs only, and 22 (10.5%) re-tested positive in either.”

were corrected as

“27 of 285 (9.5%) individuals had tested positive for SARS-CoV-2 in nasopharyngeal swab after discharged. Previously, Zhongnan Hospital has reported two (3·23%) medical staff who had tested positive after discharged. In a study of 209 discharged patients by Tang et al, 9 (4.3%) were re-tested positive in throat swabs only, 13 patients (6.2%) were re-tested positive in anal swabs only, and 22 (10.5%) were re-tested positive in either.”

6.[Line 203- “The incidence rate of our study is representative due to the diverse characteristics of the study population”. What characteristics are the authors talking about?]

【Response】: We apologize for this confusion. We tried to express that the population was representative as we have included the majority of COVID-19 patients in Guangzhou, an international city, and there were no restrictions other than age.

【Change】

Line 205-209, the statements of “The heterogeneity of these results is probably due to the disparities in the sample size and population characteristics. The incidence rate of our study is representative due to the diverse characteristics of the study population.” were corrected as “The study by Zhongnan Hospital merely included medical staff and the Third People’s Hospital of Shenzhen had only enrolled 49.4% (209/423) COVID-19 patients diagnosed in Shenzhen. The incidence rate of our study was more representative since we have included the majority (75.6%, 285/377) of COVID-19 patients in the international city: Guangzhou, with no restrictions other than age.”

7.[The author’s need to provide a justification or their view on why the patients who were negative at time of discharge after first hospitalization became positive? Is there any limitation of the test (in view of the Limit of detection) or is there a possibility that these might be case of reinfection?]

【Response】: Thank you for this kind guidance. As the reviewer mentioned, the phenomenon of RP events may due to the limitation of the sensitivity of RT-PCR testing. If RT-PCT false negative rate is 30%, then even if it is tested twice, a false negative rate of 9% would remain. Compared with NRP patients, RP patients’ prolonged time for Negative-conversing may be a reason that they were more likely to have retested positivity after discharge.

【changes】

Line 221, “Thus, we concluded that RP events are more likely caused by false negative RT-PCR tests before discharging.” was added.

8.[The justification produced to rule out reinfection completely is not reasonable, understanding that many cases now come as asymptomatic and knowing that the antibodies might have some effect on the virus the re-infected patients might present with mild symptoms, the authors can presume that these were not re-infection cases but the study methodology is not sufficient to rule out the same.]

【Response】: Thanks for the reviewers’ valuable comment, we have added discussion to this point. In our view, the retested positivity in the current study unlikely reflect reinfection. Since it’s a common practice for COVID-19 patients to have another a period of self-isolation (at least 2 weeks) after initial discharging (we have supplemented this in methodology), it is quite unlikely that the RP patients contacted other infectious Covid-19 patients during the period of self-isolation. And these RP patients showed no obvious clinical symptoms or disease progression. Therefore, we believe that the RP patients in this study were more likely to be false negative at discharge. It’s quite true as the Reviewer mentioned that we could hardly rule out all re-infection in RP patients, as COVID-19 patients from many countries don’t have to undergo a period of self-isolation (at least 2 weeks) after initial discharging.

【changes】

1. Line 89, “According to the COVID-19 management routine of Guangzhou Eighth People's Hospital, after initial discharging, COVID-19 patients have to undergo a period of isolation (within 15 days after discharge, it is unlikely to have a RP after this time) in the hospital or at home.” was added.

2. Line 213-215, “Most patients in the current study showed no obvious clinical symptoms or disease progression indicated by laboratory and CT findings which showed that these RP patients unlikely had reinfection.” was corrected as “Retested positivity unlikely reflect reinfection, since most RP patients in current study showed no obvious clinical symptoms or disease progression indicated by laboratory and CT findings and did not contact with other infectious patients.”

**Response to Reviewer 3:**

1.[the characteristic of the populations are identified as the cause of discrepancies with previous studies on the same subject, but the authors do not detail which characteristic my affect the difference? Is the average age different? Is the ethnicity different? (line 203-204)

-I do not find 285 and 209 (lines 202-203) to be so different as sample sizes and the authors did not detail which population characteristics differ among the three studies. Can the authors comment on this with more detail?]

【Response】: Through the Reviewer’s comment, we found that the statement of “diverse characteristics” was confusing. We tried to express that the population was representative as we have included the majority of COVID-19 patients in Guangzhou, an international city, and there were no restrictions other than age.

【Change】

Line 205-209, the statements of “The heterogeneity of these results is probably due to the disparities in the sample size and population characteristics. The incidence rate of our study is representative due to the diverse characteristics of the study population.” were corrected as “The study by Zhongnan Hospital merely included medical staff and the Third People’s Hospital of Shenzhen had only enrolled 49.4% (209/423) COVID-19 patients diagnosed in Shenzhen. The incidence rate of our study was more representative since we have included the majority (75.6%, 285/377) of COVID-19 patients in the international city: Guangzhou, with no restrictions other than age.”

2.[How does this number of redetectable positives compare with the specificity of the rt-pcr test they used?]

【Response】: The RP rate in this study is 9.5%, which, following simplistic statistics, suggests a false negative rate of around 30% for a single test (0.308×0.308=0.095). This is in line with the false negative rate of RT-PCR used in this study.

3.[Since some of the authors are directly responsible for performing the nasal-throat swab on patients, could they comment on how the way the nasal-throat swab is performed might affect (if at all) their results? Would a sputum-based test impact in any way on their results?]

【Response】: In the process of collection, we had encountered the patients with swelling of nasal concha, deviation of nasal septum, or tongue cannot hold down, unable to fully expose the posterior pharyngeal wall, and need to use tongue depressor tool to assist in collecting another nasal cavity, such patients are more difficult to collect and may affect the accuracy of the results. Generally speaking, the positive rate of sputum samples collected from the middle and lower respiratory tract is higher than nasal-throat swab. We usually collect sputum-based test only on patients with expectoration.

4.[ Why is there patients follow up after discharge? Is it a feature added from this study or is it common practice? If it is a feature of this study they should detail how the frequency of re-testing was decided.

Why if the discharged patients re-test positive they are re-admitted into the hospital? Is it common practice in their region? Is it a feature added by this specific study?]

【Response】In Guangzhou, it is common practice to follow up patients after discharge, and patients have to be re-admitted if they were re-tested positive. As mentioned in Line 101, the frequency of re-testing was once every two or three days during post-discharge surveillance.

【Change】

Line 89, “According to the COVID-19 management routine of Guangzhou Eighth People's Hospital, after initial discharging, COVID-19 patients have to undergo a period of isolation (within 15 days after discharge, it is unlikely to have a RP after this time) in the hospital or at home. During the surveillance, nasopharyngeal swab samples of patients were collected by staff of Guangzhou Center for Disease Control and Prevention (Guangzhou CDC) and submitted to Guangzhou Eighth People's Hospital for Reverse transcription polymerase chain reaction (RT-PCR) test. Patients with positive nucleic acid tests after discharging were diagnosed as being RP and have to be readmitted and receive further medical observation.” was added.

5.[Is there any possibility that any of the included patients has had a RP after the end of the study? They should clearly explain why this is not possible (i.e. all the patients have been discharged and checked for more than x weeks and it is unlikely to have a RP after this time).]

【Response】:

Thank you for this valuable comment, we have added this point methodology.

【Change】

This point was added in line 89, “According to the COVID-19 management routine of Guangzhou Eighth People's Hospital, after initial discharging, COVID-19 patients have to undergo a period of isolation (within 15 days after discharge, it is unlikely to have a RP after this time) in the hospital or at home.”

6.[In figure 2 maybe showing the entire distribution of the times instead of the median might be more informative. This is just a suggestion, it might also be more confusing, still it could be provided as a supplementary figure.]

【Response】:

Thanks for reviewer’s comment. We have added entire distribution of times of RP patients in the supplementary material.

【Other changes】:

1. Line 97 “viral nucleic acid testing” was corrected as “Testing process”.
2. Line 236, “This makes them more susceptible to positive result after discharged.” was added.
3. Line 409, “Supplementary Figure S1. Entire distribution of times of RP patients.” Was added.

We tried our best to improve the manuscript and made some changes in the manuscript. These changes will not influence the content and framework of the paper. Changes are marked in red in revised paper.

We appreciate for Editors/Reviewers’ warm work earnestly, and hope that the correction will meet with approval.

Once again, thank you very much for your comments and suggestions.
